# Supplementary material for: P-TRAP: a Panicle Trait Phenotyping tool
Source: BMC Plant Biol. 2013 Aug 29;13:122. doi: 10.1186/1471-2229-13-122 (PMC3848748; doi:10.1186/1471-2229-13-122)
Supplement: Additional file 7 — P-TRAP source code. The Netbeans project that contains the source code of the application. [file 1471-2229-13-122-S7.zip › javadoc.zip!/javadoc/org-bioalg-skeletonprocessor/org/bioalg/skeletonprocessor/Skeleton.html]

Skeleton (SkeletonProcessor)


JavaScript is disabled on your browser.


- Package
- Class
- Tree
- Deprecated
- Index
- Help

*org.bioalg.skeletonprocessor 1.0*

- Prev Class
- Next Class

- Frames
- No Frames

- All Classes

- Summary:
- Nested |
- Field |
- Constr |
- Method

- Detail:
- Field |
- Constr |
- Method


org.bioalg.skeletonprocessor

## Class Skeleton

- java.lang.Object
- - org.bioalg.skeletonprocessor.Skeleton

- ---

    

  ```
  public class Skeleton
  extends java.lang.Object
  ```

  Skeleton is represented as a sequence of points. The skeleton can be divided
  to different sub skeletons according to connectivity. The image can have many
  skeletons

  Author:
  :   faroq

- - ### Nested Class Summary

    Nested Classes

    | Modifier and Type | Class and Description |
    | --- | --- |
    | `static class` | `Skeleton.PIXEL_TYPE` |
  - ### Constructor Summary

    Constructors

    Constructor and Description

    `Skeleton()`

    `Skeleton(org.bioalg.imageprocessor.AImage img,
    int windowsize)`

    Classify the image pixels using a 3\*3 window to calculate the neighbors
    of a pixel if they are 1, it is an end point, >=2 an intersection , and a
    slap otherwise

    `Skeleton(java.util.ArrayList<java.awt.Point> points)`

    Create a skeleton form a set of points.
  - ### Method Summary

    Methods

    | Modifier and Type | Method and Description |
    | --- | --- |
    | `void` | `addEndPoint(java.awt.Point p)` |
    | `void` | `addIntersectionPoint(java.awt.Point p)` |
    | `void` | `addSlapPoint(java.awt.Point p)` |
    | `void` | `clearSpikes(double length)` Remove the spikes of a given length. |
    | `java.util.ArrayList<java.awt.Point>` | `getEndpoints()` |
    | `java.util.ArrayList<java.awt.Point>` | `getIntersections()` |
    | `int` | `getLength()` |
    | `java.awt.Point` | `getNearst(java.awt.Point p, int range)` Get the nearest point to the input point given a range. |
    | `java.util.ArrayList<java.awt.Point>` | `getNeighbors(java.awt.Point p, int neighborhoodRange)` Get the list of neighbors of a point given a range threshold. |
    | `Skeleton.PIXEL_TYPE` | `getPixelType(int[][] mat)` |
    | `Skeleton.PIXEL_TYPE` | `getPixelType(java.awt.Point p)` |
    | `Skeleton.PIXEL_TYPE` | `getPixelType(java.awt.Point p, int neighbors_size)` |
    | `java.util.ArrayList<java.awt.Point>` | `getSlaps()` |
    | `void` | `removeEndPoint(java.awt.Point p)` |
    | `void` | `removeIntersectionPoint(java.awt.Point p)` |
    | `void` | `removeSlapPoint(java.awt.Point p)` |

    - ### Methods inherited from class java.lang.Object

      `clone, equals, finalize, getClass, hashCode, notify, notifyAll, toString, wait, wait, wait`

- - ### Constructor Detail


    - #### Skeleton

      ```
      public Skeleton()
      ```


    - #### Skeleton

      ```
      public Skeleton(org.bioalg.imageprocessor.AImage img,
              int windowsize)
      ```

      Classify the image pixels using a 3\*3 window to calculate the neighbors
      of a pixel if they are 1, it is an end point, >=2 an intersection , and a
      slap otherwise

      Parameters:
      :   `img` -


    - #### Skeleton

      ```
      public Skeleton(java.util.ArrayList<java.awt.Point> points)
      ```

      Create a skeleton form a set of points.

      Parameters:
      :   `points` -
  - ### Method Detail


    - #### getLength

      ```
      public int getLength()
      ```


    - #### getEndpoints

      ```
      public java.util.ArrayList<java.awt.Point> getEndpoints()
      ```


    - #### getIntersections

      ```
      public java.util.ArrayList<java.awt.Point> getIntersections()
      ```


    - #### getSlaps

      ```
      public java.util.ArrayList<java.awt.Point> getSlaps()
      ```


    - #### addSlapPoint

      ```
      public void addSlapPoint(java.awt.Point p)
      ```


    - #### addIntersectionPoint

      ```
      public void addIntersectionPoint(java.awt.Point p)
      ```


    - #### addEndPoint

      ```
      public void addEndPoint(java.awt.Point p)
      ```


    - #### removeSlapPoint

      ```
      public void removeSlapPoint(java.awt.Point p)
      ```


    - #### removeIntersectionPoint

      ```
      public void removeIntersectionPoint(java.awt.Point p)
      ```


    - #### removeEndPoint

      ```
      public void removeEndPoint(java.awt.Point p)
      ```


    - #### getPixelType

      ```
      public Skeleton.PIXEL_TYPE getPixelType(java.awt.Point p)
      ```


    - #### getPixelType

      ```
      public Skeleton.PIXEL_TYPE getPixelType(java.awt.Point p,
                                     int neighbors_size)
      ```


    - #### getPixelType

      ```
      public Skeleton.PIXEL_TYPE getPixelType(int[][] mat)
      ```


    - #### getNeighbors

      ```
      public java.util.ArrayList<java.awt.Point> getNeighbors(java.awt.Point p,
                                                     int neighborhoodRange)
      ```

      Get the list of neighbors of a point given a range threshold.

      Parameters:
      :   `p` - the point
      :   `neighborhoodRange` - the range threshold

      Returns:


    - #### getNearst

      ```
      public java.awt.Point getNearst(java.awt.Point p,
                             int range)
      ```

      Get the nearest point to the input point given a range.

      Parameters:
      :   `p` - the point.
      :   `range` - the range


    - #### clearSpikes

      ```
      public void clearSpikes(double length)
      ```

      Remove the spikes of a given length.

      Parameters:
      :   `length` -


- Package
- Class
- Tree
- Deprecated
- Index
- Help

*org.bioalg.skeletonprocessor 1.0*

- Prev Class
- Next Class

- Frames
- No Frames

- All Classes

- Summary:
- Nested |
- Field |
- Constr |
- Method

- Detail:
- Field |
- Constr |
- Method
